# Repeated application of transcranial ultrasound maintains spatial and recognition memory in 5xFAD mice with reduction of amyloid-β burden

**DOI:** 10.1371/journal.pone.0336114

**Published:** 2025-11-12

**Authors:** Seung-Schik Yoo, Anvita Reddy, William Carroll, Kanyapat Ploypradith

**Affiliations:** Department of Radiology, Brigham and Women’s Hospital, Harvard Medical School, Boston, Massachusetts, United States of America; University of Nebraska Medical Center College of Medicine, UNITED STATES OF AMERICA

## Abstract

Pharmacological removal of amyloid beta protofibrils has emerged as a promising therapeutic strategy to delay the onset of Alzheimer’s disease (AD) symptoms. As a non-pharmacological and noninvasive alternative, transcranial application of low-intensity ultrasound through intact skull can induce convective acoustic streaming, which has been shown to enhance cerebrospinal fluid solute transport and facilitate the clearance of interstitial solutes. This has led to the development of device-based approaches aimed at removing the precursors of amyloid beta (Aβ) plaques and mitigating cognitive decline in AD. We applied non-thermal, non-cavitational ultrasound (400 kHz frequency) in a pulsed mode (75 ms pulse duration, 2 Hz repetition rate) to the hippocampal region of male 5xFAD mice for 30 minutes weekly, starting at 10 weeks of age and continuing for 15 weeks (until 6 months of age). Spatial and recognition memory performance was assessed monthly using the Y-maze spontaneous alternation (SA) and novel object recognition (NOR) tests. A control group of age-matched mice underwent the same procedures with receiving zero acoustic output. Mice subjected to transcranial ultrasound (tUS) treatment maintained both SA and NOR performance throughout the entire experimental period, whereas mice that received sham tUS exhibited a progressive decline in memory beginning at 3–4 months of age. Congo Red staining of the brain sections revealed a significant (> 40%) reduction in Aβ plaques in the sonicated group. Histological analysis confirmed that repeated ultrasound exposure did not cause any detectable tissue damage. These findings suggest that low intensity tUS may serve as a novel, noninvasive therapeutic strategy to delay the onset of AD symptoms through the reduction of Aβ burden.

## Introduction

The lymphatic system plays a critical role in maintaining homeostasis by collecting plasma and metabolic waste (e.g., proteins, cellular debris, and pathogens), and transporting them to peripheral lymphatic vessels for clearance [1]. Despite comprising a small fraction of body mass, the brain accounts for a disproportionately high level of energy consumption and generates substantial metabolic waste. This metabolic demand necessitates efficient waste clearance; however, the brain lacks parenchymal lymphatic vessels, prompting extensive investigation into the anatomical and functional characteristics of its waste clearance pathway [2–5].

Although the mechanisms underlying brain waste transport are not yet fully elucidated, current evidence suggests that solute and fluid movement across the brain is governed by a combination of processes, including convective bulk flow, diffusion, specific cellular transport mechanisms, and the secretion/filtration/absorption of cerebrospinal fluid (CSF) and interstitial fluid (ISF) [3,6]. Additionally, AQP4 channels expressed on the glial endfeet are considered to be a key determinant of bulk-flow by ‘propelling’ the CSF into the ISF space [7,8], together with arterial pulsation and the resulting pressure gradients between the arterial and venous perivascular space (PVS) [9,10], forming the basis of the ‘glymphatic’ system [7,8,11]. These pathways ultimately facilitate brain waste clearance into the systemic lymphatic circulation via the meningeal lymphatic vessels [12] or perineural routes leading to the cervical lymph modes (cLNs), a key drainage hub of the brain [6,8,13]. As one of the major pathways for brain solute transport and waste clearance, the extensive pial surface and widespread distribution of cerebral meningeal stomata enable non-diffusional, convective bulk-flow of CSF through the PVS [2,3,8].

Impaired waste clearance from the brain has been associated with aging [4,14], traumatic brain injury [7,15], stroke [16–18], idiopathic normal pressure hydrocephalus [19,20], and various neurodegenerative conditions, especially with link to Alzheimer’s disease (AD) and related dementia [5,21]. The excessive accumulation and deposition of polymerized metabolic amyloid beta (Aβ) monomers in the extracellular space is a hallmark of the AD and underpins the widely discussed ‘amyloid hypothesis’. Although recent advances in Aβ-targeting therapeutics—such as humanized monoclonal antibodies that promote the clearance of soluble Aβ protofibrils—have demonstrated efficacy in reducing amyloid burden in the brain, they showed only modest clinical benefit in slowing cognitive decline among patients with AD [22,23]. These treatments are also costly and associated with important safety concerns, including amyloid-related imaging abnormalities (ARIA), such as vasogenic edema (ARIA-E) and hemorrhages (ARIA-H), which are particularly common among APOEɛ4 gene carriers [24]. These limitations underscore the urgent need for safe, noninvasive strategies that can enhance Aβ clearance while minimizing adverse effects and supporting cognitive function in AD.

Mechanical pressure waves generated by ultrasound impart radiation force on the medium in their path, inducing convective fluidic flow—a phenomenon known as acoustic streaming. This effect has been utilized in fluid cyst detection (by identifying flow components within the fluid) [25,26], and in enhancing therapeutic drug delivery, a technique known as convection-enhanced delivery (CED) [27,28]. Transcranial ultrasound (tUS) enables noninvasive delivery of acoustic pressure waves to the brain, with control over the depth and size of sonication, and the ability to target broad brain regions [29,30]. Several groups, including ours, have shown that pulsed tUS, applied at subthermal, non-cavitational intensities that do not disrupt the blood-brain barrier (BBB) to the rodent brain, can safely enhance CSF solute transport [31] via acceleration of CSF flow through the PVS [32]. In rodent models, ultrasound application has been shown to enhance the clearance of exogenously introduced intracortical fluorescent tracers (ovalbumin with molecular weight of ~45 kDa, which are comparable in size to neuroactive Aβ oligomers), draining them into cLNs [33]. Additionally, through *in vitro* testing in porous media that mimics the dimensions of the PVS, we have identified an optimal set of sonication parameters (frequency and pulsing scheme) that maximize solute transport [34].

These discoveries led us to explore tUS as a potential noninvasive method for enhancing Aβ clearance with the goal of alleviating Alzheimer’s symptoms in a murine AD model. Given the importance of early and frequent Aβ removal—before its extracellular accumulation leads to detrimental effects, we hypothesized that weekly tUS sonication sessions (a total of 15 sessions) targeting the hippocampi of 5xFAD mice, beginning before the onset of spatial memory deficits, would delay cognitive decline and reduce Aβ plaques in the brain. Memory-related behavior was assessed using the Y-maze Spontaneous Alternation (SA) test and the Novel Object Recognition (NOR) test, conducted monthly between 3 and 6 months of age. At the end of the intervention, Congo Red staining was used to quantify Aβ plaque burden along with the histological assessment for abnormal features resulting from repeated tUS exposure.

## Methods and materials

### Animals

This study was carried out in accordance with the recommendations in the Guide for Care and Use of Laboratory Animals of the National Institutes of Health. All animal procedures and protocols (Protocol#: 2023N000180) were conducted according to the regulations and standards of Brigham and Women’s Hospital Institutional Animal Care and Use Committee (IACUC), and the study was carried out and reported in accordance with the ARRIVE guidelines (https://arriveguidelines.org/). A total of 12 5xFAD mice (all male; B6SJL Tg(APPSwFlLon, PSEN1M146LL286V)6799Vas/Mmjax, #03840, Jackson Laboratories, Bar Harbor, ME) were procured at 8 weeks of age. All mice were socially housed and *ad libitum* access to water and chow, while 12−12 hr light cycle was maintained (light on at 7 AM, off at 7 PM). The B6SJL transgenic strain (a cross between C57BL/6 and SJL) was chosen over the congenic C57BL/6 strain-based 5xFAD mice due to its earlier accumulation of Aβ_42_, observed as early as 2 months of age [35]. The strain also exhibits earlier behavioral impairments, with deficits emerging around 4 months of age [35,36].

### Experimental overview

**Fig 1:**
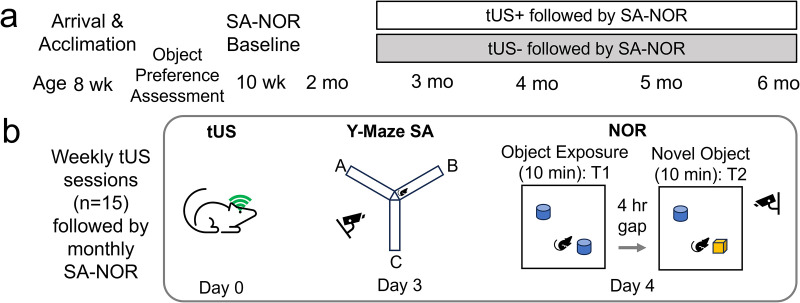
Schematics of experimental procedures. **(a)** Overall timeline of experiment (SA: Spontaneous Alternation test, NOR: Novel Object Recognition test), and **(b)** the content of weekly tUS sessions followed by monthly assessment of spatial and object recognition memory.

The experimental workflow is illustrated in **Fig 1**.

#### Animal model/group assignment/initial object preference screening.

After an acclimation period of at least three days upon arrival, the mice were randomly assigned to either the group that receive sonication (tUS+) or the one that receives sham tUS (tUS-, n = 6 each group). Individual preference bias toward the objects used in the NOR test was screened prior to intervention.

#### Baseline memory testing.

At 10 weeks of age, mice underwent initial spatial memory and novel object recognition assessments using the Y-maze Spontaneous Alternation (SA) test and the Novel Object Recognition (NOR) test (referred to as “baseline” in **Fig 1a**). Three days later, the first tUS session was administered, starting 11 weeks of age.

#### Ultrasound treatment & behavioral assessments.

Mice in the tUS+ group received 30-minute tUS session under isoflurane anesthesia (Day 0, **Fig 1b**). Although tUS itself does not induce pain, anesthesia was applied to demobilize the head during sonication. Memory performance was reassessed via SA and NOR tests on Days 3 and 4, allowing time for anesthesia recovery. The second tUS session followed one week later (at 12 weeks of age, noted as Month 3), with subsequent SA/NOR tests. Weekly tUS sessions continued until 6 months of age (15 sessions total), with monthly behavioral assessments. The tUS- group underwent the same protocol but with zero-output ultrasound. To minimize circadian/sleep rhythm confounds on memory performance and brain lymphatic clearance, tUS were administered between 11 AM and noon, while behavioral testing occurred between 10 and 11 AM.

#### Post-intervention analysis.

At 6 months of age, mice were sacrificed following the final assessment of memory behaviors, and brain tissue was harvested for histological analysis. This analysis assessed the effects of tUS on brain tissue and quantified the number of Aβ plaques per brain area (in mm^2^).

### Transcranial ultrasound

**Fig 2:**
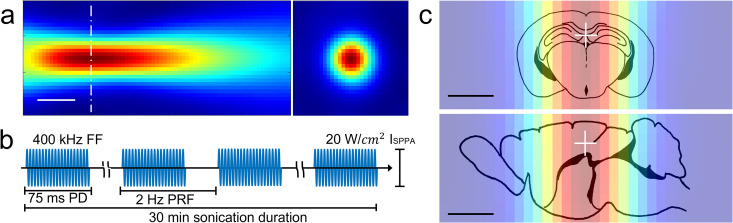
Acoustic pressure map and illustration of tUS pulsing scheme and coverage of mice brain. **(a)** Acoustic pressure map, pseudo colored from the maximum along the longitudinal (left) and transverse (right) at the focal location from dotted center line, with the arrow indicating the sonication direction. Bar = 10 mm. A graphical illustration of **(b)** ultrasound pulsing scheme and **(c)** acoustic pressure profile overlaid over the mouse brain atlas. A white cross indicates the location of pressure maximum. Bar = 5 mm.

The sonication parameter used in the present study was chosen to generate the maximal acoustic streaming effects based on our previous *in vitro* experiments [34] that examine the aqueous solute infiltration in porous media that mimic the porosity of the brain [37,38]. A 400 kHz transducer (WS100-0.5-P38, Ultran Group, State College, PA, USA) was actuated by a sinusoidal electrical waveform (function generator: 33500B, Keysight, Santa Rosa, CA, USA), amplified using a linear power amplifier (240L, Electronics and Innovations, Rochester, NY, USA) with impedance matching. The acoustic pressure field generated by the transducer was mapped and calibrated in a degassed water tank, as described previously [39,40]. The acoustic focal profile, defined by the full-width-at-half-maximum (FWHM) pressure, is outlined by the black dotted line (scale bar = 10 mm), forming an elongated ellipsoidal shape measuring 9 mm in diameter and 57 mm in length. Although the maximum pressure occurred 24 mm from the transducer’s exit plane, the pressure level remained relatively uniform within a small mice brain anatomy (**Fig 2a**). Ultrasound was applied in a pulsed manner, with a pulse duration (PD) of 75 ms and 2 Hz pulse repetition frequency (PRF) at spatial peak pulse average intensity (I_SPPA_) of 20 W/cm^2^ for 30 minutes. Considering 15% duty cycle, spatial peak temporal average intensity [I_SPTA_] was 3 W/cm^2^ (pulsing scheme illustrated in **Fig 2b**). The maximum rarefactional pressure was 770 kPa, yielding a mechanical index (MI) of 1.2, which is well below the MI threshold of 1.9 used in most ultrasound imaging applications [41,42]. Without the use of intravenously introduced microbubbles (MBs), the pressure level does not disrupt the BBB [43].

The mice were anesthetized with isoflurane (3% for induction, 1–2% for maintenance at a flow rate of 1 L/min) and placed in a prone position on a lab-built stereotactic frame. A 37°C warming pad (Gaymar, Orchard Park, NY) was placed underneath to maintain body temperature. The body posture was maintained throughout the experiment across all animals to minimize variations in associated brain lymphatic clearance [44]. Fur over the head was removed using depilation lotion, as needed. The transducer was positioned along the midline, with the acoustic focus at 2 mm depth, 1.5 mm rostral to the interaural line, ensuring sonication of most of the hippocampal regions (see **Fig 2c**). A compressible acoustic coupling hydrogel was molded in inverted cone shape (9% w/v polyvinyl alcohol in distilled water, 18–6-hour freeze–thaw cycles) and was placed between the transducer and the scalp. Acoustic hydrogel (Parker Lab, Fairfield NJ) was applied to all interfaces. Respiratory rate (RR), heart rate (HR), and SpO_2_ were monitored every 5 minutes (SurgiVet, Smiths Medical, Keene, NH). To minimize confounding effects from variations in anesthetic depth on brain lymphatic clearance [9,10], isoflurane concentration was adjusted to maintain ~100 breaths/min, ~ 370 bpm heart rate, and 88–92% SpO_2_. After recovery, the mice were returned to a holding cage.

### Spontaneous alternation test

The mice were moved to a holding cage in the lab and allowed to acclimate to the lighting condition (~9.5 Lux; MT-912, Urceri, Shenzhen, China). A Y-maze (each arm ~ 40 cm length, ~ 8 cm width, ~ 20 cm height, with a center triangular space, each side 8 cm) was used for the SA test. The mouse was allowed to explore the maze freely for 5 minutes, while a video camera (Brio, Logitech, Lausanne, Switzerland) recorded the sequence of entries into each arm. The percent successful spontaneous alternation was calculated as the number of non-repeating sequential arm entries divided by the total number of arm entries minus two [45]. Following the SA test, the animal was moved to an NOR test chamber (approximately 40 x 40 x 40 cm, white plastic chamber) without any objects in it and allowed to acclimate for 10 minutes. After each session, the maze was cleaned with 70% ethanol and dried.

### Object preference evaluations

The NOR test is used to assess recognition memory by presenting the mice with a familiar and a novel object [46]. The time spent with each object was measured to evaluate object recognition in rodents. Five pairs of different objects, each having similar size and complexity in shape, were used in this study (labeled A–E, see S1 Fig). Before testing, the mice were acclimated to a temporary holding cage and an empty arena for 10 minutes three consecutive days. Then, the presence of innate object preferences was assessed for each animal. During each test, the mouse was placed at the center of the arena, and a pair of objects were positioned in opposite quadrants (e.g., upper left and bottom right corners). The mouse was allowed to explore the objects for 10 minutes before being returned to the holding cage. A video camera above the arena recorded animal behavior, and three rators who were blinded to the sequences of testing and experimental conditions, recorded exploration time on each object, with “exploration” defined as approaching the object within 1 cm and engaging with it (climbing, sniffing, or touching). Time spent on top of the object (after climbing) and dismounting from it was excluded. To minimize memory effects, the preference tests were conducted over 5 days, with two pairs of non-overlapping objects tested per day. Object pair placement and sequence were randomized.

A Discrimination Index (DI) was calculated as the difference in exploration times between the two objects, divided by the sum of the exploration times (e.g., for objects A and B: (t_A_ - t_B_)/ (t_A_ + t_B_)). A DI of 0 indicates no preference, while |DI| = 1.0 represents a complete preference for one object. A |DI| greater than 0.15 indicates a strong preference bias [46,47], so object pairs with a DI greater than this threshold were not used in the subsequent NOR test to a specific animal. As shown in S1 Table, while individual mice showed some degrees of object-specific preference, the group-averaged |DI| was below 0.15, indicating no group-based bias toward any specific object. Only object pairs with neutral preference (|DI| ≤ 0.15) were used in the subsequent NOR test for each animal.

### Novel object recognition test

The NOR test consisted of two sessions. In the initial object exposure session (T1), the mouse was placed in the chamber with two identical objects and allowed to explore them for 10 minutes. Afterward, the animal was returned to its holding cage. Four hours later, a second testing session (T2) was conducted in the same manner as T1, except one of the identical objects (the ‘familiar object’) was replaced with a novel object that differed in shape and color (**Fig 1b**). The 4-hour interval, referred to as the ‘retention interval’, was chosen due to its maximal sensitivity toward memory impairments in 5xFAD mice [48]. After completing both sessions, the animals were returned to their home cage. A pair of novel objects for each monthly trial was randomly selected from unbiased object pairs (|DI| ≤ 0.15, See S1 Table), counterbalanced for magnitude (i.e., accounting for both positive and negative DI values), while avoiding sequential repetition of the same pair. The quadrant location of a novel object was randomized and balanced between the pairs to minimize the potential confounding effects from associated spatial memory. The arena and objects were cleaned with 70% ethanol and dried between the sessions. The DI (i.e., for novel object and familiar object: (t_novel_ – t_familiar_)/ (t_novel_ + t_familier_)) and Recognition Index (D3) were calculated (i.e., t_novel_/ (t_novel_ + t_familier_)). The rators were all blinded to the experimental conditions. In normal phenotypic mice, DI is ~ 0.3 while D3 is ~ 0.6–0.7 [46,49]. The total exploration time, which represents general physical activity, was also recorded and compared between groups.

### Histological analysis

Upon completion of the experiments, all animals were deeply anesthetized using 4% isoflurane anesthesia to alleviate suffering during sacrifice and subjected to transcardial perfusion with 4% paraformaldehyde in phosphate-buffered saline (PBS) for extraction of the brain. The extracted brain underwent immersion fixation for approximately 24 hours for histological analysis. Since the acoustic waves were delivered to a broad brain region encompassing the hippocampus (as illustrated in **Fig 2C**), plaque load was mainly quantified in the coronal sections along the center of the beam profile. The brains were coronally sectioned, with two non-contiguous sections sampled from the sonication site (~1.5 mm from the interaural line), spanning from 2.86 to −0.08 mm relative to the interaural line. Hematoxylin and eosin (H&E) staining was used to assess general tissue morphology and structural abnormalities, while vascular amyloid fibril (VAF) staining was employed to evaluate the absence of ischemic injury. Aβ plaques were stained with Congo red. The entire field of view of each coronal section was imaged at ×20 magnification using a robotic light microscope (Evos FL Auto2, Thermo Fisher, Waltham, MA) and assembled into a mosaic image. Using ImageJ software, the sectional image was divided into 70 ~ 80 square grids (each 33^2^ pixels), and Aβ plaques were counted by two rators (blinded to the experimental conditions) and averaged for each animal from three categorized regions – neocortical, hippocampal, and the remaining allocortical areas (denoted as ‘Neo’, ‘Hippo’, and ‘Allo’, respectively). The morphological criteria for the plaques included both compact, intensely-stained core and lightly-stained diffuse plaque patterns with irregular borders.

## Results

### Body weight and key vital signs during tUS procedure

We used two-tailed tests throughout the study where a priori hypothesis on specific direction of measures cannot be justified. The body weights of the animals were statistically equivalent between the experimental groups across all timepoints (S2 Fig, two-tailed t-test, minimum p = 0.67 at 10-wk of age). The key vital signs (RR, HR, and SpO_2_) were statistically equivalent between the groups across the sessions – (1) RR; mean ± standard deviation, tUS + : tUS- = 99.0 ± 1.0: 99.3 ± 1.6, (2) HR; tUS + : tUS- = 365.5 ± 3.1: 366.1 ± 3.0, and (3) SpO_2_; tUS + :tUS- = 89.6 ± 0.6: 90.1 ± 0.6 (all two-tailed t-test, p > 0.4 in RR, p > 0.36 in HR, p > 0.1 in SpO_2_). These measures were also equivalent across the sessions within the groups – (1) RR; Multi-way ANOVA, F(14,70) = 0.77, p = 0.69 and F(14,70) = 0.50, p = 0.92, for tUS+ and tUS- respectively, (2) HR; F(14,70) = 0.45, p = 0.95 and F(14,70) = 0.31, p = 0.99, and (3) SpO_2_; F(14,70) = 0.82, p = 0.65: F(14,70) = 1.23, p = 0.27.

### Y-Maze spontaneous alternation (SA) test

**Fig 3:**
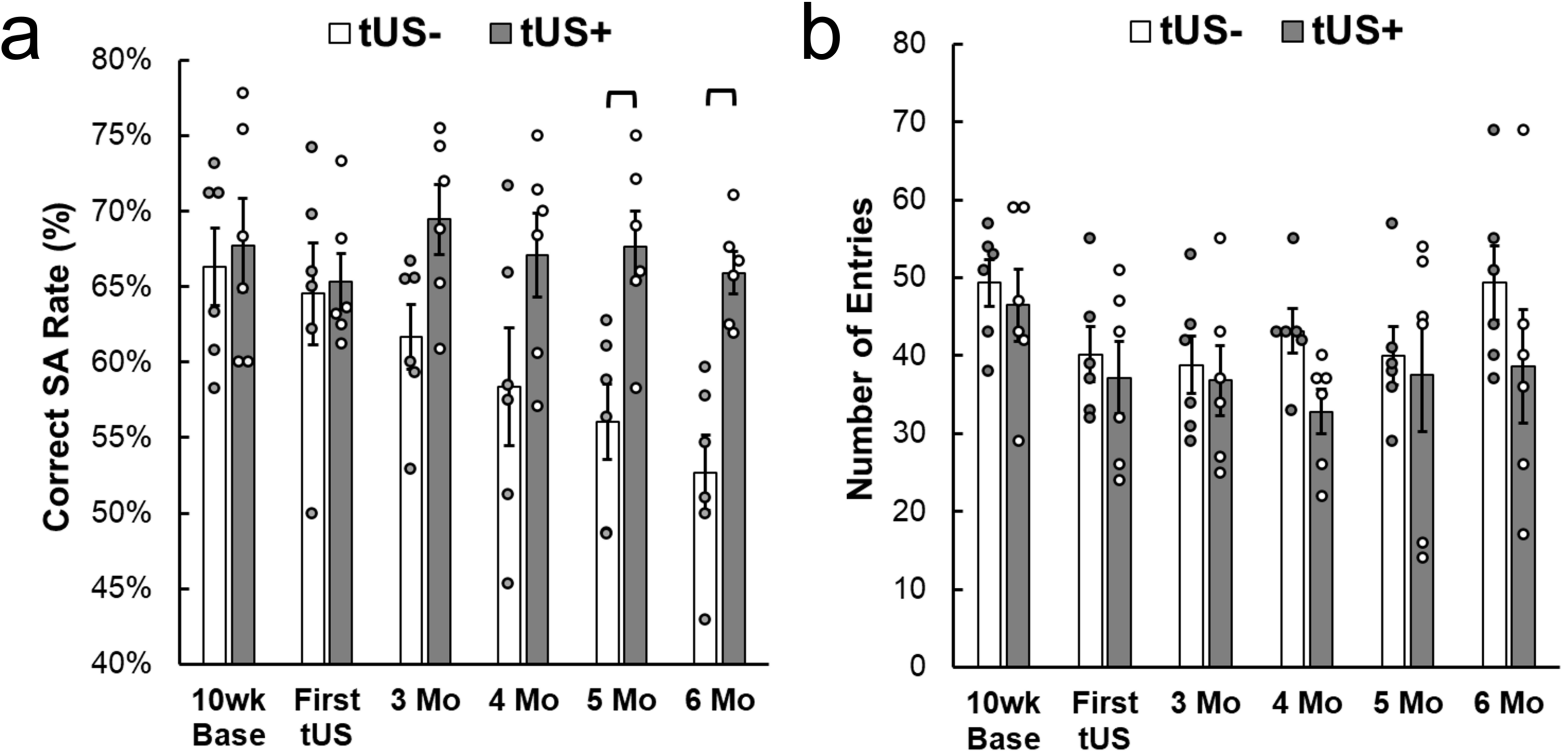
Data from spatial memory performance from Y-maze SA test. **(a)** Group-averaged successful SA % and **(b)** number of arm entries in the Y-maze across the duration of experiment (n = 6/group, circles indicate individual data). Brackets: p < 0.05, Bonferroni-corrected. Error bars: standard error.

Detailed time-wise successful SA rate (%, mean ± SEM) and outcome of statistical analysis are shown in **Fig 3a** and S2 Table. A two-way repeated-measures ANOVA (RM ANOVA, mixed-effects model) revealed a significant main effect of Group (χ^2^(1) = 9.03, p = 0.0027), a significant main effect of Time (χ^2^(5) = 12.39, p = 0.030), and a significant Group × Time interaction (χ^2^(5) = 12.76, p = 0.026). Post hoc one-tailed t-test comparisons, justified by a priori hypothesis that tUS would preserve the SA rate relative to tUS– condition, showed no difference between groups at baseline (10 weeks) or immediately after the first tUS session. By 3–4 months, the tUS– group exhibited a trend toward reduced SA compared to the tUS+ group (3 Mo: p = 0.017 uncorrected, p = 0.100 Bonferroni; 4 Mo: p = 0.049 uncorrected, p = 0.296 Bonferroni). At later timepoints, these differences reached significance: at 5 months (mean difference = −11.6%, p = 0.0037 uncorrected, p = 0.022 Bonferroni) and 6 months (mean difference = −13.2%, p = 0.00046 uncorrected, p = 0.0027 Bonferroni), with the tUS– group showing markedly lower SA performance relative to tUS + . RM ANOVA analysis regarding the number of Y-maze arm entries (**Fig 3b**; detailed data analysis in S2 Table) revealed no significant Group × Time interaction (χ^2^(5) = 2.61, p = 0.76) and no main effect of Group (χ^2^(1) = 2.54, p = 0.11), including non-significant trend for Time (χ^2^(5) = 9.66, p = 0.086). Post hoc one-tailed comparisons confirmed that the number of entries was comparable between groups at all timepoints (Bonferroni corrected p-value > 0.747). Together, these findings indicate that while both groups began with comparable performance, longitudinal decline in SA, observed in the tUS– group, was not observed in the tUS+ group, supporting a protective effect of ultrasound intervention. Furthermore, the differences observed in SA rate were not attributable to changes in general locomotor activity.

### Novel object recognition test

**Fig 4:**
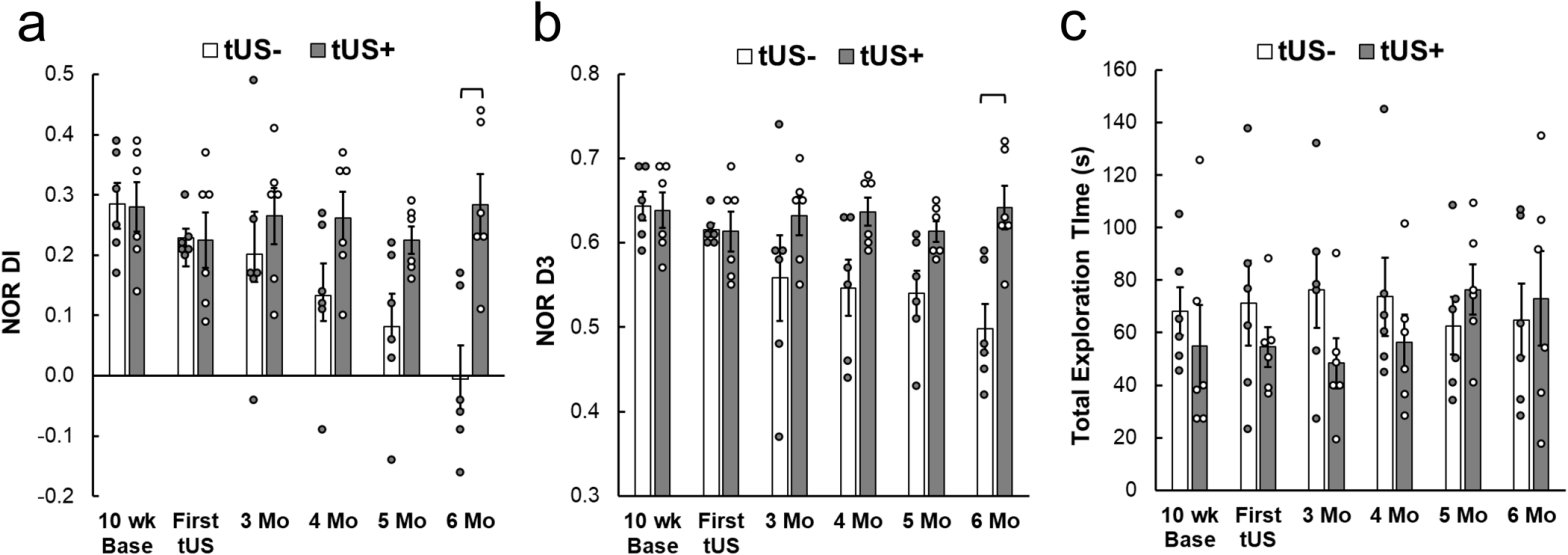
Data from object recognition memory performance from NOR test. **(a)** Group-averaged DI, **(b)** D3, and **(c)** total exploration time across the duration of experiment (n = 6/group, circles indicate individual data). Brackets: p < 0.05, Bonferroni-corrected. Error bars: standard error.

Analysis of the NOR DI (**Fig 4a**, presented in S3 Table) using a two-way RM ANOVA revealed a significant main effect of Group (χ^2^(1) = 6.78, p = 0.0092), a significant main effect of Time (χ^2^(5) = 13.36, p = 0.0202), and a significant Group × Time interaction (χ^2^(5) = 17.73, p = 0.0033). Post hoc one-tailed time-wise t-test comparisons, based on the priori expectation that tUS would preserve recognition memory, confirmed no baseline differences between groups (10 wk). Divergence emerged over time: at 4 and 5 months the tUS– group showed a trend toward lower DI values compared to tUS+ (p = 0.044 and p = 0.017 uncorrected; Bonferroni-adjusted p = 0.265 and p = 0.101, respectively). By 6 months, the decline in the tUS– group became statistically significant (mean difference = −0.29, p = 0.0016 uncorrected; Bonferroni-adjusted p = 0.0098), while the tUS+ group maintained stable positive DI scores across all sessions. These findings demonstrate that animals under tUS+ condition retained recognition memory performance over time, whereas tUS– animals exhibited a progressive decline culminating in significant impairment at 6 months of age.

Similarly, Analysis of the NOR D3 index (**Fig 4b**, S3 Table) using a two-way RM ANOVA revealed a significant main effect of Group (χ^2^(1) = 7.06, p = 0.0079) and a significant Group × Time interaction (χ^2^(5) = 16.01, p = 0.0068), with Time showing a trend-level effect (χ^2^(5) = 10.73, p = 0.057). Post hoc one-tailed comparisons, based on the hypothesis that tUS would preserve recognition performance, showed no group differences at baseline (10 wk, After FUS1) or at 3 months. At 4 and 5 months, tUS– animals exhibited a trend toward reduced D3 values compared to tUS+ (uncorrected p = 0.018 and 0.017, Bonferroni-adjusted p = 0.109 and 0.100, respectively). By 6 months, this divergence reached statistical significance (mean difference = −0.14, uncorrected p = 0.0020, Bonferroni-adjusted p = 0.012), with tUS– animals showing a clear decline while tUS+ animals maintained stable D3 values across all timepoints. Together with findings on DI, these results demonstrate that tUS prevented the progressive decline in NOR performance observed in untreated animals, with preservation evident at 6 months of age.

Analysis of total object exploration time (**Fig 4c**, S3 Table) revealed no significant Group × Time interaction (χ^2^(5) = 8.31, p = 0.14), no main effect of Group (χ^2^(1) = 0.49, p = 0.49), and no main effect of Time (χ^2^(5) = 1.37, p = 0.93). Across all sessions, tUS+ and tUS– animals spent comparable amounts of time exploring objects (mean exploration range: 48–76 s), with no systematic differences between groups or across timepoints. These findings indicate that recognition memory divergence was not attributed to variations in total exploration time.

### Histological analysis and aβ plaque burden

**Fig 5:**
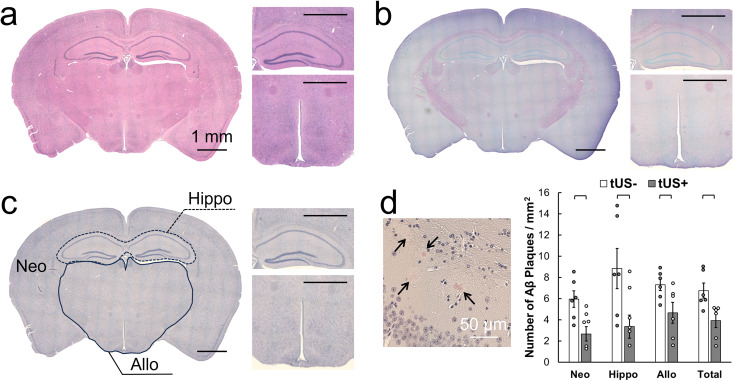
Histological data and aβ plaque burden from experimental mice. An example of **(a)** H&E, **(b)** VAF, and **(c)** Congo Red staining from the sonicated brain slice, with the illustration outlining the brain areas of interest, i.e., neocortical (Neo), hippocampal (Hippo), and remaining allocortical areas (Allo). Bars = 1 mm. **(d)** [left] An example of Congo-red stained Aβ plaques (in arrows), [right] the average number of Aβ plaques/mm^2^ across the different brain regions. 6 mice/group. Brackets: p < 0.05. Error bars: standard error.

An exemplary histology from the sonicated brain section is shown in Fig 5a–c. H&E staining showed no signs of structural/vascular abnormalities or damage from the sonicated brain area. There was no indication of ischemic injury, as confirmed by the VAF staining. Application of tUS was associated with a consistent reduction in amyloid plaque burden across the brain regions (**Fig 5d**, see S4 Table for individual data). In the neocortex, plaque density was significantly lower in the tUS+ group compared to tUS– (3.4 ± 1.6 vs. 6.0 ± 1.9 plaques/mm^2^; all one-tailed t-test, t = 2.52, p = 0.016). A similar effect was observed in the hippocampus, where plaque counts were reduced from 8.8 ± 4.7 to 4.6 ± 2.7 plaques/mm^2^ (t = 1.94, p = 0.044). The remaining allocortex likewise showed a significant decrease in the plaque burden with tUS (7.3 ± 1.3 vs. 4.7 ± 2.4 plaques/mm^2^; t = 2.34, p = 0.024). When analyzed across all regions combined, the total plaque density was markedly reduced in tUS+ animals relative to unsonicated controls (3.9 ± 1.6 vs. 6.8 ± 1.7 plaques/mm^2^; t = 2.84, p = 0.0087), by more than 42%. Collectively, these findings indicate that tUS exposure significantly reduced Aβ plaque accumulation across multiple brain regions. There was no difference in total Aβ plaque density between the left and right brain hemisphere (in tUS+ condition, Left: Right = 3.9 ± 1.6: 3.9 ± 1.9 plaques/mm^2^, two-tailed paired t-test, p = 0.93; in tUS-, Left: Right = 6.9 ± 2.1: 6.6 ± 1.4 plaques/mm^2^, p = 0.49), which indicated that there was no preferential hemispheric clearance of aβ affected by the sonication.

## Discussion

Although exact mechanisms underlying brain solute transport and clearance remain under investigation, monoclonal antibody-based pharmacological treatment aimed at reducing Aβ profibril in early phase of AD suggest that clearance of unwanted metabolic byproducts, including Aβ monomers and oligomers, may play a crucial role in delaying disease progression. However, the lack of effective non-pharmacological therapies to promote CSF and interstitial solute clearance, particularly those targeting oligomeric precursors of Aβ plaque formation, represents a critical gap in the treatment of AD.

Device-based techniques that enhance solute clearance from the brain have emerged, for example, multisensory (visual and auditory) stimulation has been shown to improve brain lymphatic clearance in an AD mouse model via regulation of arterial pulsatility [50]. Ultrasound-based approaches to enhance brain lymphatic clearance also emerged, via temporary opening of the BBB by inducing stable cavitation of intravenously injected MBs combined with the application of ultrasound to the brain [51,52]. This effect presumably stems from enhanced solute transport facilitated by increased level of perivascular and interstitial fluid exchange [53,54]. However, the technique carries potential risks, including cavitation-related tissue damage (*e.g.*, hemorrhage) [55,56] and modified local hemodynamic properties, along with unknown long-term effects from repeated BBB disruption [57–59]. Others have demonstrated that tUS sonication, administered below the threshold for BBB disruption without administering MBs, can enhance CSF and intrathecally injected drug transport in normal phenotype mice [60]. More recently, Park and colleagues reported that daily, 2hr-long applications of gamma frequency tUS (3 ms PD, 40 Hz PRF, given 200 ms-long duration, every 1 sec) to the brain of six-month-old 5xFAD mice for 2 weeks have shown to increase EEG gamma power, while reducing insoluble Aβ_42_ in pre- and infra-limbic cortex, along with reduction in number of Aβ tangles [61]. These findings support the potential use of tUS for the treatment of AD. However, the efficacy of (1) repeated low intensity tUS, (2) given in a duration that can be applicable to human use (under 1 hour), (3) started in early phase of disease onset, on delaying memory deficit and reducing Aβ plaque load remain unexplored. In the present study, we administered weekly tUS, each 30-min long, to the hippocampal regions of 5xFAD mice, starting prior to the time that is known to manifest both pathological and behavioral symptoms, for 15 weeks. The intervention remarkably preserved spatial and recognition memory up to six months of age while significantly reducing Aβ burden compared to the unsonicated control. Histological analysis revealed no abnormalities beyond the expected Aβ accumulation.

Leinenga and colleagues recently reported that weekly low-intensity ultrasound (1 MHz, 10 ms PD, and 10 Hz PRF, 6 sec in total duration per treatment) over 8 weeks in an AD mouse model (APP23 mice) improved memory behavior of the mice compared to the untreated group, as assessed by the active place avoidance (APA) test [62]. Despite the improvement in the memory behavior, they did not observe any changes in Aβ load from their study. Although differences exist in study design (e.g., sonication parameters, AD mouse strain, and animal age), reduced Aβ burden observed from the present study suggests that a more intensive—longer (30 min per session) and more often (weekly over 15 weeks)—tUS intervention, which started before significant Aβ accumulation might have contributed to the difference in findings. Extending the monitoring and intervention period (e.g., > 9 months), as well as adjusting the timing of treatment onset, and frequency of treatments (for example, more than 2 times per week, even daily), will provide further insights into the dose-dependent effects of tUS on behavioral and histopathological changes associated with AD.

We found that Aβ burden, defined by the number of Congo Red-labeled plaques per area, were reduced in the tUS+ group. Because Congo Red primarily labels late-stage, compact fibrillar Aβ plaques, these findings do not fully capture the effects of tUS on other forms of Aβ aggregation, such as soluble monomers, oligomers, or protofibrils that arise earlier in pathological development. Furthermore, direct evidence of increased Aβ protein drainage into the deep cervical lymph nodes (dCL) has yet to be identified and quantified. The degree of Aβ protein drainage to the dCL can be addressed by performing immunoassays to measure its Aβ levels following a single ultrasound session in AD mice models. Additionally, because Aβ plaques were quantified only in sonicated brain regions and limited to visible macroscopic deposits, a more comprehensive assessment—such as incorporating thioflavin-S staining with epifluorescence microscopy across a broader brain volume (e.g., including the olfactory bulbs and cerebellum)—will be needed. In this regard, techniques such as SWATH-MS (Sequential Window Acquisition of All Theoretical Mass Spectra) or direct immunoassays (e.g., northern blotting) can also provide information on the types of Aβ fragments (e.g., Aβ_40/42_ oligomers, protofibril, or even tangles) affected by the tUS-mediated solute clearance. In addition to these histological and molecular assays, *in vivo* imaging methods, such as positron emission tomography (PET) utilizing amyloid-sensitive radiopharmaceuticals (e.g., Floretaben or Florpetapir), may help elucidate the relationship between tUS treatments and time-progression of Aβ clearance.

The current study utilized sonication parameters known to induce convective bulk flow in fluidic media via acoustic streaming effects. Similar parameters have been shown to promote cerebrospinal fluid (CSF) and interstitial solute transport and clearance from the brain without eliciting neural stimulation [33,34]. However, contributions from other tUS-related mechanisms, such as upregulation of TRPV4-AQP4 and the associated enhancement of glymphatic influx and clearance [63] or enhanced brain lymphatic transport associated with direct activation of neuronal tissue [61,64,65], cannot be entirely ruled out. Future studies could benefit from using AQP knockout mice or AQP blockers, such as 2-(nicotinamide)-1,3,4-thiadiazole (TGN-020), or use of assessment of immediate early gene (IEG) expression associated with neuronal activity (e.g., c-fos or Egr1) after sonication, to assess the relative contributions of different mechanisms in ultrasound-induced Aβ clearance. We also note that mice in this study were anesthetized to enable reliable sonication, which introduces a potential confound since both anesthetic agent and depth can modulate glymphatic/lymphatic dynamics and thereby influence clearance. For example, light anesthesia with agents such as isoflurane, ketamine/xylazine, or dexmedetomidine has been reported to enhance glymphatic influx and clearance compared to wakefulness, likely through slow-wave–like neural activity and reduced noradrenergic tone that expand the interstitial space and promote CSF–ISF exchange [66]. In contrast, deeper or prolonged isoflurane anesthesia may diminish vascular pulsatility, a key driver of CSF–ISF transport. To mitigate this confound, we conducted experiments under a light, stable anesthetic plane, confirmed by equivalent vital signs (respiratory rate, heart rate, SpO₂) between the experimental groups [67]. Nevertheless, residual confounding effects cannot be excluded, underscoring the need for future studies in awake mice using implanted or wearable ultrasound devices.

We acknowledge other limitations of this exploratory, proof-of-concept study. Given the known sex-dependent differences in AD progression [68,69], inclusion of both male and female subjects will be essential in future work. Another limitation is the absence of wild-type (WT) control mice. Although 10-week-old 5xFAD mice are generally considered pre-symptomatic, age-matched WT cohorts are needed to account for variability in the onset of behavioral symptoms, which may differ across breeding colonies, laboratories, and experimental conditions. To broaden applicability, future studies should also evaluate the technique across other AD mouse strains (e.g., APP, 3xTg).

We also note that the present tUS approach may preferentially enhance clearance of extracellular Aβ and have limited impact on intracellular tau aggregates because acoustic streaming primarily influences extracellular compartments. However, given the close interplay between Aβ and tau accumulation, for example, via Aβ-induced hyperphosphorylation of tau or via neuroinflammation [70,71], it is plausible that tau pathology could also be modulated by tUS. This hypothesis warrants further investigation in murine models exhibiting tau accumulation, for example, in 3xTg mice. Furthermore, while tUS was applied before the onset of memory impairment in this study, the optimal timing of treatment remains unclear. It is also unknown whether tUS treatment could reverse AD pathology once the disease has already progressed. These knowledge gaps demand further studies that will evaluate the effects from different treatment onset timing on reversing/delaying the disease progression.

The acoustic beam created by transducer in the present study passed through large portion of the mice brain. Since Aβ pathology affects multiple brain regions, achieving broader brain coverage in humans will be necessary for future clinical translation. This highlights the need for a new ultrasound hardware configuration capable of delivering sonication covering entire brain volume in humans while accommodating more frequent interventions in non-clinical settings (aiming home-based treatment), likely to be achieved by using non-focal beam mode combined with multi-array ultrasound transducer configurations.

Given that the accumulation of Aβ plaques and fibrils is a hallmark feature of AD [70,72,73], tUS represents a noninvasive and repeatable strategy to enhance the clearance of Aβ precursors prior to plaque formation, and may offer elegant and unprecedented means to delay the onset of AD dementia or slow the progression of cognitive decline. tUS could also be used as an adjunct maintenance therapy to reduce cumulative side effects or mitigate risk in patients particularly vulnerable to ARIA. From a scientific standpoint, successful demonstration of tUS-mediated Aβ clearance would provide compelling additional evidence for the role of the brain’s waste clearance system in AD pathophysiology. This, in turn, can lay the groundwork for a broader therapeutic paradigm that incorporates the enhancement of endogenous clearance mechanisms as a strategy for AD prevention and treatment.

Beyond applications to AD, tUS has the potential to benefit the treatments of a wide spectrum of neurological disorders associated with impaired brain lymphatic function and inefficient metabolic waste clearance. These include stroke, where disrupted fluid exchange contributes to neuroinflammation and secondary injury [74,75]; multiple sclerosis, in which inflammation and demyelination can impair astrocyte function and disrupt the perivascular clearance system, potentially hindering the removal of inflammatory mediators and metabolic waste [76,77]; neurodegenerative proteinopathies such as Parkinson’s disease; and traumatic brain injury, which are characterized by the accumulation of misfolded proteins like α-synuclein and tau [7,75]. By enhancing convective flow and lymphatic clearance, tUS could support the removal of pathogenic proteins. Moreover, improving brain lymphatic function through tUS may help mitigate the buildup of metabolic byproducts (e.g., lactate) associated with sleep deprivation [78,79].

Repeated ultrasound sonication in AD mouse models, using parameters shown to enhance solute clearance via acoustic streaming, preserved memory function while reducing Aβ plaques. Collectively, these findings suggest that tUS may serve as a noninvasive, non-pharmacological strategy for AD treatment. However, given the small sample size and the exclusive use of male mice, the results should be interpreted as proof of concept rather than broadly generalizable conclusions. Further research is urgently needed to refine the technique for preclinical applications involving larger, sex-balanced cohorts and to clarify the mechanisms by which harmful brain solutes are cleared.

## Supporting information

S1 Fig Five objects (A through E) used for the NOR. The ruler in the middle is 5 cm-long.
(DOCX)

S2 Fig Body weight of mice measured over the duration of experiment and range of physiological data across the verum/sham tUS conditions. Error bars: standard error. (a) Body weight measured from baseline (10-wk of age) to 6 months of age (circles indicate individual data, n = 6 each group) (b) Box plots of group average distribution of respiratory rate, heart rate, and SpO_2_ across 15 tUS treatment sessions (n = 6 each group). × indicates the mean value.
(DOCX)

S1 Table Results of initial preference scores and p-value comparing each pair of objects (A through E) between two groups (t-test, two-tailed). (DOCX)

S2 Table Group data on successful SA % and number of arm entries in the Y-maze cross the duration of experiments (mean ± SEM), including one-tailed post hoc comparisons (tUS– vs. tUS+), showing Bonferroni corrected p-value (P_Corr_) at each timepoint. (DOCX)

S3 Table Group data on NOR DI, NOR D3, and total exploration time (mean ± SEM), including one-tailed post hoc comparisons (tUS– vs. tUS+) showing Bonferroni corrected p-value (P_Corr_) at each timepoint. (DOCX)

S4 Table The number of Aβ plaques/mm^2^ across the different brain regions–neocortical (Neo), hippocampal (Hippo), and remaining allocortical (Allo) areas– across all animals (n = 6 per group). (DOCX)
