# Supplementary figures and images for: Repeated application of transcranial ultrasound maintains spatial and recognition memory in 5xFAD mice with reduction of amyloid-β burden
Source: PLoS One. 2025 Nov 12;20(11):e0336114. doi: 10.1371/journal.pone.0336114 (PMC12611139; doi:10.1371/journal.pone.0336114)

**S1 Figure** Five objects (A through E) used for the NOR. The ruler in the middle is 5 cm-long


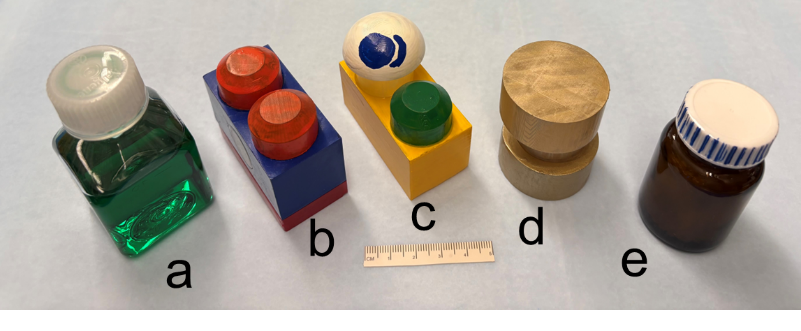

Supplement: S1 Fig — The ruler in the middle is 5 cm-long. (DOCX) [file pone.0336114.s001.docx]
