# Supplementary material for: Repeated application of transcranial ultrasound maintains spatial and recognition memory in 5xFAD mice with reduction of amyloid-β burden
Source: PLoS One. 2025 Nov 12;20(11):e0336114. doi: 10.1371/journal.pone.0336114 (PMC12611139; doi:10.1371/journal.pone.0336114)
Supplement: S2 Fig — Error bars: standard error. (a) Body weight measured from baseline (10-wk of age) to 6 months of age (circles indicate individual data, n = 6 each group) (b) Box plots of group average distribution of respiratory rate, heart rate, and SpO2 across 15 tUS treatment sessions (n = 6 each group). × indicates the mean value. (DOCX) [file pone.0336114.s002.docx]

**S2 Figure** Body weight of mice measured over the duration of experiment and range of physiological data across the verum/sham tUS conditions. Error bars: standard error. (a) Body weight measured from baseline (10-wk of age) to 6 months of age (circles indicate individual data, n=6 each group) (b) Box plots of group average distribution of respiratory rate, heart rate, and SpO_2_ across 15 tUS treatment sessions (n=6 each group). × indicates the mean value.

**
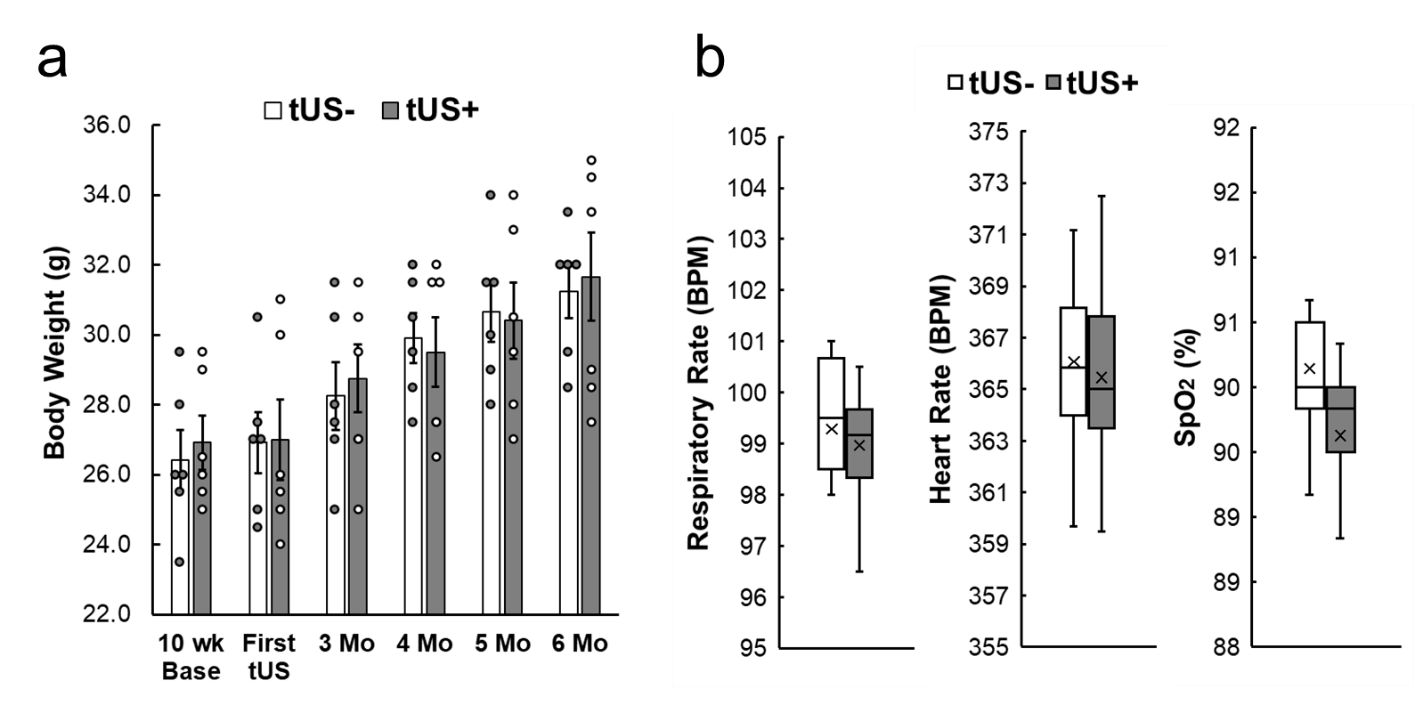
**
