# Supplementary material for: Repeated application of transcranial ultrasound maintains spatial and recognition memory in 5xFAD mice with reduction of amyloid-β burden
Source: PLoS One. 2025 Nov 12;20(11):e0336114. doi: 10.1371/journal.pone.0336114 (PMC12611139; doi:10.1371/journal.pone.0336114)
Supplement: S1 Table — (DOCX) [file pone.0336114.s003.docx]

**S1 Table** Results of initial preference scores and p-value comparing each pair of objects (A through E) between two groups (t-test, two-tailed).

|  |  | **AB** | **AC** | **AD** | **AE** | **BC** | **BD** | **BE** | **CD** | **CE** | **DE** | Mean |
| --- | --- | --- | --- | --- | --- | --- | --- | --- | --- | --- | --- | --- |
| tUS- | 1 | -0.04 | 0.29 | -0.10 | 0.17 | 0.16 | -0.07 | 0.13 | -0.07 | 0.21 | 0.06 | 0.07 |
|  | 2 | -0.20 | -0.19 | 0.15 | -0.34 | 0.15 | 0.15 | 0.35 | -0.11 | 0.26 | 0.39 | 0.06 |
|  | 3 | -0.12 | 0.11 | 0.19 | 0.20 | 0.14 | 0.27 | 0.07 | -0.08 | 0.06 | -0.21 | 0.06 |
|  | 4 | 0.08 | -0.15 | 0.21 | 0.11 | 0.09 | -0.20 | -0.11 | -0.03 | 0.18 | -0.78 | -0.06 |
|  | 5 | 0.47 | 0.00 | 0.16 | -0.02 | 0.02 | 0.23 | 0.34 | -0.01 | -0.18 | 0.18 | 0.12 |
|  | 6 | 0.05 | -0.03 | 0.03 | -0.16 | -0.19 | 0.02 | -0.13 | -0.10 | 0.04 | -0.25 | -0.07 |
|  | Mean | 0.04 | 0.00 | 0.11 | -0.01 | 0.06 | 0.07 | 0.11 | -0.07 | 0.09 | -0.10 |  |
|  |  |  |  |  |  |  |  |  |  |  |  |  |
| tUS+ | 1 | 0.02 | 0.07 | -0.19 | 0.14 | 0.13 | -0.25 | 0.09 | 0.05 | -0.04 | -0.25 | -0.02 |
|  | 2 | -0.21 | -0.19 | 0.02 | 0.24 | 0.27 | 0.02 | 0.48 | -0.14 | -0.01 | -0.34 | 0.02 |
|  | 3 | -0.21 | 0.32 | 0.06 | 0.06 | -0.17 | -0.01 | -0.02 | -0.20 | -0.11 | 0.10 | -0.02 |
|  | 4 | 0.09 | -0.23 | 0.22 | 0.26 | 0.24 | 0.44 | 0.18 | -0.36 | -0.06 | 0.07 | 0.09 |
|  | 5 | 0.03 | 0.11 | -0.05 | -0.14 | -0.06 | 0.33 | 0.21 | -0.09 | 0.32 | -0.12 | 0.05 |
|  | 6 | -0.06 | 0.05 | 0.16 | 0.22 | 0.08 | 0.16 | 0.08 | 0.02 | 0.01 | 0.09 | 0.08 |
|  | Mean | -0.06 | 0.02 | 0.04 | 0.13 | 0.08 | 0.11 | 0.17 | -0.12 | 0.02 | -0.07 |  |
|  |  |  |  |  |  |  |  |  |  |  |  |  |
|  | *p-value* | 0.40 | 0.86 | 0.40 | 0.23 | 0.81 | 0.72 | 0.59 | 0.41 | 0.42 | 0.89 | 0.97 |
