# Supplementary material for: Repeated application of transcranial ultrasound maintains spatial and recognition memory in 5xFAD mice with reduction of amyloid-β burden
Source: PLoS One. 2025 Nov 12;20(11):e0336114. doi: 10.1371/journal.pone.0336114 (PMC12611139; doi:10.1371/journal.pone.0336114)
Supplement: S2 Table — (DOCX) [file pone.0336114.s004.docx]

**S2 Table** Group data on successful SA % and number of arm entries in the Y-maze cross the duration of experiments (mean ± SEM), including one-tailed post hoc comparisons (tUS– vs. tUS+), showing Bonferroni corrected p-value (P_Corr_) at each timepoint.

| **SA Rate (%)** | | | **Number of Arm Entries** | | |  |
| --- | --- | --- | --- | --- | --- | --- |
| Time | tUS- | tUS+ | P_Corr_ | tUS- | tUS+ | P_Corr_ |
| 10 wk Base | 66.3±2.6 | 67.7±3.1 | 1 | 49.3±3.0 | 46.5±4.7 | 1 |
| First tUS | 64.5±3.4 | 65.3±1.9 | 1 | 40.2±3.5 | 37.2±4.6 | 1 |
| 3 Mo | 61.7±2.2 | 69.4±2.3 | 0.1 | 38.8±3.7 | 36.8±4.5 | 1 |
| 4 Mo | 58.4±3.9 | 67.1±2.8 | 0.296 | 43.2±2.9 | 32.8±2.9 | 0.089 |
| 5 Mo | 56.1±2.5 | 67.6±2.4 | 0.022 | 40.0±3.8 | 37.5±7.3 | 1 |
| 6 Mo | 52.7±2.5 | 65.9±1.4 | 0.003 | 49.3±4.8 | 38.7±7.3 | 0.747 |
