# Supplementary material for: Repeated application of transcranial ultrasound maintains spatial and recognition memory in 5xFAD mice with reduction of amyloid-β burden
Source: PLoS One. 2025 Nov 12;20(11):e0336114. doi: 10.1371/journal.pone.0336114 (PMC12611139; doi:10.1371/journal.pone.0336114)
Supplement: S3 Table — (DOCX) [file pone.0336114.s005.docx]

**S3 Table** Group data on NOR DI, NOR D3, and total exploration time (mean ± SEM), including one-tailed post hoc comparisons (tUS– vs. tUS+) showing Bonferroni corrected p-value (P_Corr_) at each timepoint.

|  | **DI** | | | **D3** | | | **Total Exploration Time (sec)** | | |
| --- | --- | --- | --- | --- | --- | --- | --- | --- | --- |
| Time | tUS- | tUS+ | P_Corr_ | tUS- | tUS+ | P_Corr_ | tUS- | tUS+ | P_Corr_ |
| 10 wk Base | 0.28±0.04 | 0.28±0.04 | 1 | 0.64±0.02 | 0.64±0.02 | 1 | 68.1±9.1 | 55.0±15.6 | 1 |
| First tUS | 0.23±0.01 | 0.22±0.05 | 1 | 0.62±0.01 | 0.61±0.02 | 1 | 71.3±16.3 | 54.6±7.6 | 1 |
| 3 Mo | 0.20±0.07 | 0.26±0.05 | 1 | 0.56±0.05 | 0.63±0.02 | 0.649 | 76.3±14.5 | 48.4±9.6 | 0.418 |
| 4 Mo | 0.13±0.05 | 0.26±0.04 | 0.265 | 0.55±0.03 | 0.64±0.02 | 0.109 | 73.7±14.9 | 56.3±10.7 | 1 |
| 5 Mo | 0.08±0.05 | 0.22±0.02 | 0.101 | 0.54±0.03 | 0.61±0.01 | 0.1 | 62.6±11.1 | 76.4±9.7 | 1 |
| 6 Mo | 0.00±0.05 | 0.28±0.05 | 0.010 | 0.50±0.03 | 0.64±0.03 | 0.012 | 64.7±13.9 | 73.1±18 | 1 |
