# Supplementary material for: Repeated application of transcranial ultrasound maintains spatial and recognition memory in 5xFAD mice with reduction of amyloid-β burden
Source: PLoS One. 2025 Nov 12;20(11):e0336114. doi: 10.1371/journal.pone.0336114 (PMC12611139; doi:10.1371/journal.pone.0336114)
Supplement: S4 Table — (DOCX) [file pone.0336114.s006.docx]

**S4 Table** The number of Aβ plaques/mm^2^ across the different brain regions–neocortical (Neo), hippocampal (Hippo), and remaining allocortical (Allo) areas– across all animals (n=6 per group)

| **tUS-** | | | | | | | |
| --- | --- | --- | --- | --- | --- | --- | --- |
|  | 1 | 2 | 3 | 4 | 5 | 6 | Mean ± SEM |
| Neo | 8.54 | 5.80 | 7.56 | 6.10 | 4.20 | 3.48 | 5.95 ± 0.79 |
| Hippo | 4.42 | 3.45 | 13.80 | 14.76 | 8.28 | 8.30 | 8.84 ± 1.91 |
| Allo | 5.38 | 7.10 | 7.92 | 8.33 | 8.91 | 6.19 | 7.30 ± 0.55 |
| Total | 5.82 | 5.87 | 9.04 | 8.72 | 6.31 | 4.82 | 6.67 ± 0.65 |

| **tUS+** | | | | | | | |
| --- | --- | --- | --- | --- | --- | --- | --- |
|  | 1 | 2 | 3 | 4 | 5 | 6 | Mean ± SEM |
| Neo | 1.49 | 3.81 | 5.28 | 3.75 | 4.54 | 1.26 | 3.36 ± 0.67 |
| Hippo | 1.56 | 3.27 | 7.02 | 4.19 | 2.91 | 8.56 | 4.59 ± 1.09 |
| Allo | 1.60 | 6.45 | 4.08 | 7.44 | 6.24 | 2.24 | 4.67 ± 0.98 |
| Total | 1.54 | 4.59 | 5.11 | 5.08 | 4.91 | 2.26 | 3.92 ± 0.65 |
